# Nutritional Trends in Cystic Fibrosis: Insights from the Italian Cystic Fibrosis Patient Registry

**DOI:** 10.3390/jcm13133652

**Published:** 2024-06-22

**Authors:** Donatello Salvatore, Rita Padoan, Annalisa Amato, Marco Salvatore, Giuseppe Campagna, on behalf of the Italian CF Registry Working Group

**Affiliations:** 1 Cystic Fibrosis Centre, Hospital San Carlo, 85100 Potenza, Italy; 2 Scientific Board, Italian CF Registry, 00100 Rome, Italy; ritaf54@gmail.com (R.P.); amatoann@libero.it (A.A.); marco.salvatore@iss.it (M.S.); gius.campagna@gmail.com (G.C.); 3 Italian Cystic Fibrosis League, Charity Programme, 00162 Rome, Italy; 4 Undiagnosed Rare Diseases Interdepartmental Unit, Istituto Superiore di Sanità, National Center Rare Diseases, 00161 Rome, Italy; 5 Department of Medical-Surgical Sciences and Translational Medicine, University of Rome Sapienza, 00161 Rome, Italy; 6 Department of Clinical and Molecular Medicine, University of Rome Sapienza, 00189 Rome, Italy

**Keywords:** cystic fibrosis, nutrition, obesity

## Abstract

**Background:** Over the past decades, efforts have been made to improve the nutritional well-being of people with cystic fibrosis (pwCF). Due to the correlation observed between nutritional indices and lung function, prevailing recommendations consistently advocate for BMI percentile goals at or above the 50th percentile in pwCF. Recent global trends show a notable increase in overweight and obese statuses among pwCF. This study aims to explore the nutritional status of Italian pwCF. **Methods:** Data from the Italian CF Patient’s Registry were analysed to assess the proportion of individuals categorized as underweight, target weight, overweight, and obese from 2010 to 2021. Patient-level comparison data from 2021 were also examined to identify the potential determinants of overweight and obesity. **Results:** Analysis spanning 2010 to 2021 reveals a decrease of approximately 40% in underweight status among adults, while the proportion of malnourished patients younger than 18 years remained stable. Conversely, there was a substantial increase of over 70% in overweight status and over 85% in obesity among adults, with minor fluctuations observed among children and adolescents. Patient factors associated with increased obesity incidence included age older than 45 years, male gender, pancreatic sufficiency, possession of at least one CFTR variant conferring residual function, ppFEV_1_ > 90, and lower prevalence of *Pseudomonas aeruginosa* colonization. **Conclusions:** Our study confirms the evolving nutritional status landscape among Italian adult pwCF, with a significant shift towards overweight and obesity over the past decade. These trends highlight the need for proactive measures within CF standards of care to adapt and address the changing needs of patients.

## 1. Introduction

Cystic fibrosis (CF) management recognizes the critical role of nutrition. Historically, weight gain has been a primary objective due to the high prevalence of malnutrition and low body weight observed in this patient population [1]. To address this, the CF Nutrition Guidelines established recommendations for target nutritional indices based on Body Mass Index (BMI) [2,3].

There exists a well-established association between Body Mass Index (BMI) and pulmonary function in CF management. Data consistently collected from various patient registries demonstrate a positive correlation between these two variables. This correlation is typically assessed through percent predicted forced expiratory volume in one second (ppFEV_1_). Notably, patients with a BMI at the 50th percentile exhibit a normal average ppFEV_1_. Interestingly, this average ppFEV_1_ continues to improve even as BMI increases into the overweight and obese categories [4,5]. Furthermore, evidence suggests that early life nutritional status has a long-term impact on pulmonary function within the paediatric CF population [6]. Conversely, undernutrition remains a significant risk factor associated with poorer pulmonary outcomes, increased colonization with Pseudomonas aeruginosa (Pa), diminished quality of life, and heightened mortality rates [7].

Clinicians involved in the care and management of patients with CF have made nutrition and weight gain a priority in their quality improvement initiatives. This focus has led to an increase in the median BMI, even surpassing the 50th percentile in many CF Centres. This trend suggests that a substantial proportion of patients with CF now fall into the overweight or obese categories [8,9]. This shift in emphasis aligns with the broader trend of rising obesity rates in the general population.

Multiple advancements have contributed to the improved outcomes observed in CF management. These advancements include earlier diagnosis, facilitated by newborn screening programs, the prompt initiation of pancreatic enzyme replacement therapy (PERT), more effective and timely nutritional and medical interventions, and, most notably, the recent introduction of CFTR modulators. The introduction of CFTR modulators, beginning with Ivacaftor in 2012 and culminating in the late 2019 approval of Elexacaftor-Tezacaftor-Ivacaftor (ETI), has revolutionized CF care. CFTR modulators have been incorporated into care programs across several countries and have demonstrated substantial benefits in both nutritional and respiratory outcomes [10]. Several international initiatives are currently estimating the efficacy and the effects of these drugs by making use of the increasing amount of data included in CF Registries, including the Italian CF Patient’s registry (ICFR).

This paper explores the evolving landscape of nutritional status in Italian people with CF (pwCF), assessing the prevalence of overweight and obese individuals within this population, and also verifying whether malnutrition still remains a problem to be addressed in pwCF.

## 2. Materials and Methods

We utilized data from the Italian CF Patient’s Registry (ICFR). ICFR collects data from 29 different Italian CF Centres; data are focussed on demography, diagnosis (including new diagnosis per year), genetics, lung function, nutrition, complication, microbiology, transplantation, and mortality. Aspects related to eligibility criteria, data collection, generalizability, and validity of the data have been comprehensively documented [4].

### 2.1. Inclusion Criteria

To ensure the relevance and integrity of our analysis, the following criteria were applied to the study population:

- Individuals with a confirmed diagnosis of CF;
- Inclusion in the ICFR for at least one year within the period spanning from 2010 to 2021.
- Patients aged two years or older at the time of inclusion.
- At least one follow-up visit during the year, with a documented height and weight measurement.
- Transplanted patients were included in the study population.

### 2.2. Study Components

Our research encompassed two key components:

1. Trends Analysis (2010–2021): ICFR has been collecting quality data since 2010. For the purposes of this study, we conducted an in-depth examination of trends in the nutritional status of the entire CF population aged two years and older. Specifically, we assessed the proportion of individuals falling into underweight, target weight, overweight, and obese categories over the period spanning from 2010 to 2021. The percentage of subjects excluded from this analysis due to missing data ranged from 19.7% in 2010 to 11.1% in 2021 for adults, and from 38.3% in 2010 to 4.3% in 2021 for children.
2. Patient Level Comparison data of the year 2021: focusing on the year 2021, we compared factors between adults aged 18 and older and children and adolescents aged 2–17.9 years, who were classified as overweight and obese and those who were not. This analysis aimed to identify potential determinants of overweight and obesity within the CF patient population. The percentages of subjects excluded from this analysis due to missing data were 11.1 for adults and 4.3 for children.

### 2.3. Ethical Considerations

This study on anonymous patient data was approved by the Scientific and Steering Committees of the ICFR on 26 June 2023 (approval code: SC0623P). Data collected within the ICFR and used for this study purposes, are in compliance with the indication and the approval of the Italian National Ethical Committee; informed consent was obtained from eligible individuals or their legal representatives, as per the established protocols of the ICFR.

### 2.4. Variables and Data Analysis

1. Weight Group Classification: to categorize individuals based on their nutritional status, we utilized annualized BMI percentiles or values, depending on the age of the patient. For individuals younger than 18 years, we employed BMI percentiles, while those aged 18 and above were classified based on BMI values in kg/m^2^. The weight group designations were as follows [11]:
  ∘ BMI < 18.5 kg/m^2^ or <5th percentile (underweight);
  ∘ BMI 18.5–24.9 kg/m^2^ or 5th–<85th percentile (target weight);
  ∘ BMI 25–29.9 kg/m^2^ or 85th–<95th percentile (overweight);
  ∘ BMI > 30 kg/m^2^ or >95th percentile (obese).
2. Demographic and Clinical Factors: we considered a range of demographic and clinical patient-level factors to gain a comprehensive understanding of the factors associated with nutritional status in CF patients. These factors included:
  ∘ Sex;
  ∘ Age;
  ∘ Genotype;
  ∘ Diagnosis by Newborn Screening (NBS);
  ∘ Annualized percent predicted forced expiratory volume at one second (ppFEV_1_);
  ∘ Pancreatic status, defined by proxy according to the utilization of pancreatic enzyme replacement therapy (PERT);
  ∘ Diabetes status;
  ∘ Colonization by *Pseudomonas aeruginosa* (*Pa*);
  ∘ Use of inhaled hypertonic saline (HS) and inhaled antibiotics;
  ∘ Use of CFTR modulators.

### 2.5. Methodology for Weight Group Classification

The assignment of individuals to weight groups was carried out based on their annualized BMI percentile or value, depending on their age. To calculate annualized measures, we used the programs written in SAS language available on the site https://www.cdc.gov/growthcharts/computer_programs.htm, accessed on 11 October 2023. For individuals aged less than 18 years, weight group classification relied on BMI percentile, while individuals aged 18 or older were categorized based on BMI values in kg/m^2^.

### 2.6. Statistical Analyses

Continuous variables presented as mean ± standard deviation (SD) and categorical variables are shown as absolute frequency and percentage.

Symmetry/normality of BMI and BMI percentile was evaluated by Shapiro–Wilk test and checking Q–Q plot.

The associations between BMI and BMI percentile classes with respect to the categorical variables analysed in Table 1 and Table 2 were evaluated by X^2^ or Cochran–Armitage trend test.

Bubble plots were realised for the weight category rates of paediatric and adult patients from 2010–2021. A *p* value < 0.05 was considered statistically detectable.

Statistical analysis was performed using SAS version 9.4 TS Level 1 M8 and JMP PRO version 17 (SAS Institute, Cary, NC, USA).

## 3. Results

In 2021, the cohort of ICFR patients consisted of 3332 individuals aged 18 years and above and 2015 aged between 2 and 17.9 years. The analysis of the CF population over the review period from 2010 to 2021 revealed notable trends in nutritional status.

### 3.1. Nutritional Trends over Time

**Figure 1:**
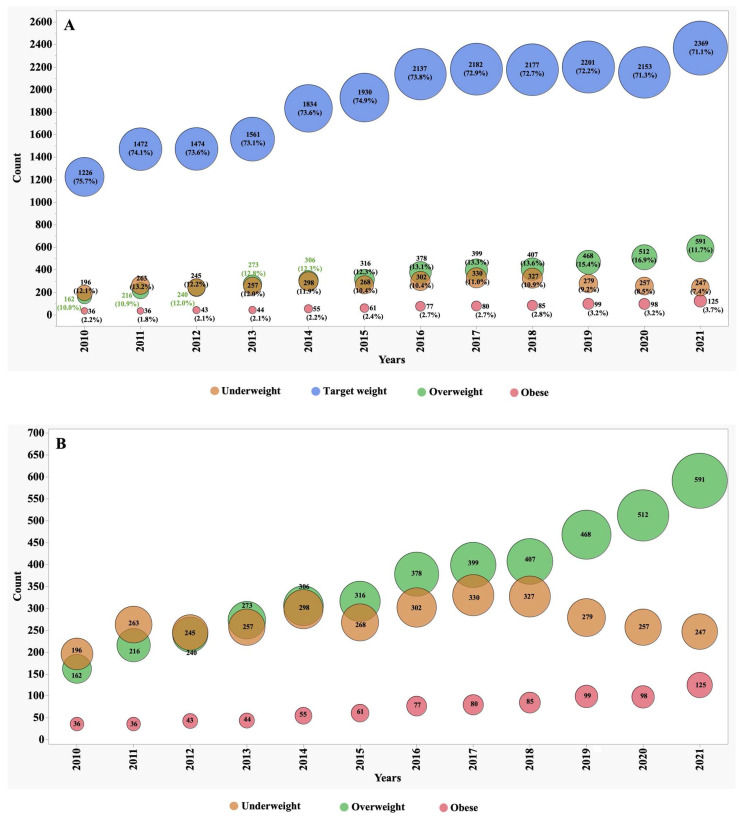
Distribution of weight groups of adult patients (older than 18 years) from 2010–2021: (**A**) all four weight groups; (**B**) focus on first three weight groups.

**Figure 2:**
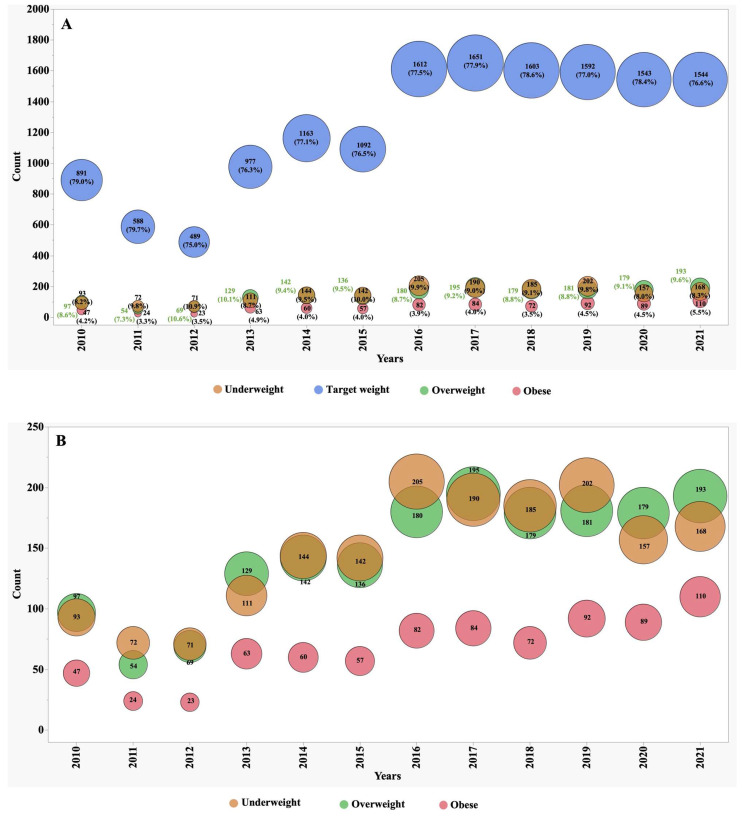
Distribution of weight groups in paediatric patients (2–17.9 years) from 2010–2021: (**A**) all four weight groups; (**B**) focus on first three weight groups.

Between the years 2010 and 2021, we observed significant shifts in the numbers, proportions, and frequency of underweight, overweight, and obese patients (Figure 1 and Figure 2). In 2010, individuals classified as underweight accounted for 12.1% of the adult CF population and 8.2% of the paediatric subgroup. However, these percentages exhibited distinct patterns of change over the observation period. In the adult CF population, the percentage of underweight individuals, after remaining stable during the initial half of the observation period, markedly decreased to 7.4% by 2021. Conversely, in the paediatric group, the percentage of underweight individuals remained relatively stable, with minor variations, and stood at 8.3% in 2021.

During the first five years of observation, approximately 74% of adult patients fell within the target BMI range. However, this percentage experienced a slight decline to 71% during the subsequent five years. This decline was primarily driven by an increase of over 70% in the number of patients categorized as overweight, surging from 10% to 17%, and over 85% those classified as obese, which rose from 2% to 3.7%.

In contrast, the paediatric subgroup exhibited only minor variations in the proportion of subjects falling within the target BMI z-score, overweight, and obese categories. These nuanced fluctuations suggest that the nutritional trends in the paediatric CF population were relatively stable during the observation period.

It is relevant to observe that since 2016, the proportion of both adults and children achieving their target weight has progressively increased.

### 3.2. Patient Characteristics Associated with Different Nutritional Status (Patient Level Comparison on Data of the Year 2021)

Table 1 and Table 2 offers a comprehensive summary of patient-level factors, categorized by nutritional status. Our analysis revealed statistically significant differences in nutritional status rates across various patient-level factors.

Within the adult population, certain subgroups exhibited significantly higher rates of overweight and obesity. Males showed higher rates—with 21.9% classified as overweight and 4.2% as obese—compared to females—who exhibited 13.1% overweight and 3.3% obesity. Furthermore, individuals aged over 35, particularly those aged 45 or older, had overweight rates reaching 23.7% and obesity at 6.9% (Table 1). With regard to the effect of genotypes, those carrying at least one allele with a CFTR mutation with *residual function (RF)* displayed overweight at 24.8% and obesity at 7.5%. In terms of lung function, individuals with a ppFEV_1_ exceeding 90% exhibited overweight at 24.1% and obesity at 5.4%. Patients prescribed CFTR modulators displayed a different distribution of weight categories compared to untreated subjects. Patients under treatment had higher rates of individuals within the target weight category (76.6% vs. 65.3% in adults), along with fewer overweight and obese subjects (overall 16.0% in treated adults vs. 27.1% in untreated, respectively). Those not prescribed PERT exhibited overweight rates of 24.9%, with obesity at 9.4% whereas patients with pancreatic insufficiency exhibited 14.6% overweight and 1.2% obese, respectively. Lastly, rates of overweight and obesity were higher among subjects with lower prevalence of *Pa* colonization and lower burden of inhaled treatments (hypertonic saline and antibiotics).

Among younger subjects, subgroups with higher rates of overweight and obesity were characterized by ppFEV_1_ exceeding 90%, the absence of PERT, and the presence of *RF* CFTR mutations in the genotype (Table 2). Children also displayed a distinct distribution of weight categories concerning the use of CFTR modulators. Specifically, the target weight category included 80.5% of treated children compared to 75.3% of untreated children. Conversely, fewer overweight and obese subjects were observed in the treated group, with 11.4% of treated children falling into these categories as opposed to 16.3% in the untreated group.

In terms of underweight individuals, within the adult subgroup, associations were found with female sex, age younger than 35, ppFEV_1_ levels lower than 70%, especially those below 40%, and the presence of CF-related diabetes (CFRD). Conversely, in the younger group, notable factors associated with underweight were ppFEV_1_ levels lower than 70%, particularly below 40%, and the presence of CFRD.

## 4. Discussion

Our study reveals a noteworthy and concerning trend: a consistent rise in the prevalence of overweight and obese individuals among the Italian CF adult population over the past decade. However, it is crucial to note that this trend is not extended to the paediatric age group. Despite significant efforts within CF programs to reduce the number and proportion of underweight patients, the most striking shift has been the substantial increase in the number of individuals entering the overweight or obese categories, a pattern not observed among paediatric patients. These divergent trajectories in nutritional status are complex and merit thorough exploration and consideration within the Italian (and international) CF community.

A key finding of our study pertains to patient factors associated with overweight and obesity among individuals with CF. Particularly notable are the so-called *RF* CFTR mutations. This observation aligns with prior studies from the ICFR and the European CF Registry (EU CF Registry), which also demonstrated better nutritional status in individuals with *RF* mutations compared to those homozygous *for F508del* or carrying CFTR *minimal function* mutations. The lower prevalence of pancreatic insufficiency, lower sweat chloride levels at diagnosis, and better lung function at all age groups all reflect a relatively milder disease, even though the progression of lung disease is still evident [12,13].

Adult patients categorized as overweight or obese are more likely to be male, older than 45 years, exhibit pancreatic sufficiency, possess better lung function, and receive treatment with CFTR modulators. Additionally, they are less prone to CFRD and *Pa* colonization, and have, as a logical consequence, a minor burden of inhaled treatments. Similarly, overweight and obese children were characterized by normal lung function, pancreatic sufficiency, and *RF* CFTR mutations. These observations suggest a milder overall disease severity in overweight and obese CF patients, consistent with the higher prevalence of less severe CFTR mutations, particularly Class IV–V mutations (i.e., *RF* mutations).

The association between CFTR modulators use and weight gain merits substantial attention and further investigation. It is worth noting that the impact of the highly efficient recent modulator ETI, commercially available in Italy since July 2021, is not yet fully evaluable in our study. Several proposed mechanisms underlie this association, encompassing changes in exocrine pancreas function, alterations in energy expenditure, shifts in body composition, and variations in intestinal microbiota [14]. To comprehend these intricate mechanisms and their collective impact on nutritional status, vigilant monitoring of nutritional parameters is essential. Such monitoring will be pivotal in deciphering the precise mechanisms of action and facilitating the provision of tailored guidance and care to patients with CF.

A notable observation in our study is the presence of a sex disparity in the prevalence of overweight and obesity, a phenomenon documented in previous years within the general population in Italy [15]. Our analysis of Italian pwCF reveals a somewhat analogous pattern. Specifically, we observe that the proportion of obese men exceeds that of obese women within the CF population. This sex-specific variation underscores the importance of considering sex as a potential modifier of nutritional status in CF. Our findings align with recent data from the US, Canadian, and German CF Registries, where comparable trends of increasing overweight and obese status have been documented [5,16,17]. Simultaneously, there has been a noticeable decline in the prevalence of individuals classified as having normal weight or being underweight. However, it is crucial to acknowledge that European CF Registry data indicate divergent outcomes across various European nations [18]. These regional disparities underscore the complex interplay of factors influencing nutritional status in CF patients, including genetics, healthcare practices, access to therapies, and cultural or environmental influences.

Additionally, we emphasize that the observed nutritional improvements seem largely limited to the adult patient population. Data from the paediatric subgroup of our CF population indicate that in the last decade, the relative percentages of underweight, target, overweight, and obese children remained relatively stable. However, it is important to note that the eligibility for ETI for children aged 12 and above, and subsequently for children aged 6–11, was obtained in Italy in July 2021 and September 2022, respectively. Only about 200 subjects over 12 years old with a % predicted FEV_1_ below 40 received elexacaftor/tezacaftor/ivacaftor through a managed access program in Italy before July 2021, the approval date of this CFTR modulator. Given this limited number, we believe it is highly unlikely that this event significantly impacted our overall results.

Therefore, only 43.4% of the adult subgroup and 22.8% of the paediatric subgroup were treated by CFTR modulators at the time of our analysis; therefore, we are unlikely to be able to observe the effects of the highly efficient modulators at the present time. In Germany, one year of treatment with ETI reduced the percentage of underweight children from 28.9% to 17.9%, suggesting a significant impact in the near future [17]. Nonetheless, ensuring adequate nutrition for children with CF remains a priority.

Our study has several limits. It focuses on the Italian population, so the findings may not be generalizable to other countries due to potential differences in genetics, healthcare practices, access to therapies, and cultural or environmental factors. Moreover, this kind of study finds associations between factors like CFTR mutations, lung function, and weight status. However, it cannot definitively prove that these factors cause weight gain. Another limit is represented by the fact that our study does not explore how diet and exercise habits might influence weight changes in this population.

Conversely, this study boasts several strengths that contribute valuable insights. The study analyses data from a large patient population, allowing for robust statistical analysis and generalizable trends within the Italian CF population. The research sheds light on the concerning rise in overweight and obesity among adults with CF, a potential consequence of improved treatments but not previously well documented. The study design allows for comparison between adult and child patient groups, revealing a different trend in weight status between the two and explores the sex-based disparity in weight gain observed, which can help tailor future interventions. Finally, the findings on weight gain and CFTR mutations align with previous studies from other registries, strengthening the overall observations.

In this complex landscape characterized by a wide and dynamic spectrum of nutritional conditions, new challenges are emerging for CF care teams. Considering that obesity, dyslipidaemia, aging, and hyper caloric diets, enriched in fat, sugar, and salt, are well-established risk factors for cardiovascular disease (CVD), it is prudent to anticipate a potential increase in CVD cases among persons with CF in the coming years, particularly in those who are pancreatic sufficient [19]. Consequently, it is imperative that future investigations, including case studies, observational research, and longitudinal studies, are conducted within this specific patient cohort to substantiate this hypothesis. There is also the need to integrate the data collection of the CF registers with information on the appearance of further adult complications, such as hypertension, or coronary artery disease [20]. Conversely, it is essential to maintain measures to improve nutrient intake and digestion and to adopt the appropriate nutritional intervention to paediatric pwCF, where undernutrition is even now evident [21,22]. The push towards the improvement of nutrition from CFTR modulators in younger patients will be investigated in the coming years.

## 5. Conclusions

In conclusion, our study underscores and confirms the evolving scenery of nutritional status in the Italian adult CF population, with a pronounced shift towards overweight and obesity over the past decade. These trends, which are likely to extend to younger CF patients in the near future, necessitate proactive measures within CF standards of care to adapt and address the changing needs of patients. The association between obesity and milder CFTR mutations, along with the intriguing link between CFTR modulator use and weight gain, enriches our understanding of CF pathophysiology. Future research endeavours should focus on elucidating the mechanisms underlying these observations to enhance the management and care of individuals living with CF.

## Figures and Tables

**Table 1:** Characteristics of weight categories in adult patients (age 18 and older).

Parameter	Overall n = 3332	Underweight (n = 247) (% = 7.41) n (%)	Target Weight (n = 2369) (% = 71.10) n (%)	Overweight (n = 591) (% = 17.74) n (%)	Obese (n = 125) (% = 3.75) n (%)	*p*
**Sex**						**<0.0001**
Male	1742	90 (5.17)	1197 (68.71)	382 (21.93)	73 (4.19)	
Female	1590	157 (9.87)	1172 (73.71)	209 (13.14)	52 (3.27)	
**Age**						**<0.0001**
18 to 35	1907	184 (9.65)	1398 (73.31)	278 (14.58)	47 (2.46)	
35 to 45	715	43 (6.01)	498 (69.65)	145 (20.28)	29 (4.06)	
45+	710	20 (2.82)	473 (66.62)	168 (23.66)	49 (6.9)	
**ppFEV_1_**						**<0.0001**
<40	230	43 (18.7)	159 (69.13)	26 (11.3)	2 (0.87)	
40 to 70	976	89 (9.12)	735 (75.31)	130 (13.32)	22 (2.25)	
70 to 90	864	56 (6.48)	632 (73.15)	143 (16.55)	33 (3.82)	
90+	1139	46 (4.04)	758 (66.55)	274 (24.06)	61 (5.36)	
**Inhaled HS**						**0.02**
No	1833	139 (7.58)	1270 (69.28)	343 (18.71)	81 (4.42)	
Yes	1496	108 (7.22)	1098 (73.4)	247 (16.51)	43 (2.87)	
**Inhaled antibiotic**						**<0.0001**
No	1745	113 (6.48)	1166 (66.82)	371 (21.26)	95 (5.44)	
Yes	1585	134 (8.45)	1203 (75.9)	219 (13.82)	29 (1.83)	
**Diabetes**						**<0.0001**
No	2135	137 (6.42)	1473 (68.99)	422 (19.77)	103 (4.82)	
Yes	802	70 (8.73)	601 (74.94)	119 (14.88)	12 (1.5)	
***Pa* colonization**						**<0.0001**
No	1529	92 (6.02)	1050 (68.67)	306 (20.01)	81 (5.3)	
Yes	1299	109 (8.39)	971 (74.75)	195 (15.01)	24 (1.85)	
**PERT**						**<0.0001**
No	1012	40 (3.95)	625 (61.76)	252 (24.9)	95 (9.39)	
Yes	2319	207 (8.93)	1744 (75.2)	339 (14.62)	29 (1.25)	
**CFTR Modulator**						**<0.0001**
*Treated*	1446	*108 (7.47)*	*1109 (76.69)*	*210 (14.52)*	*19 (1.31)*	
*Not treated*	1886	*139 (7.37)*	*1260 (66.81)*	*381 (20.20)*	*106 (5.62)*	
**Genotype**						
F/F	693	61 (8.8)	536 (77.34)	92 (13.28)	4 (0.58)	**<0.0001**
F/G	62	3 (4.84)	44 (70.97)	11 (17.74)	4 (6.45)	0.62
F/RF	312	13 (4.17)	194 (62.18)	80 (25.64)	25 (8.01)	**<0.0001**
F/MF	875	70 (8)	680 (77.71)	119 (13.6)	6 (0.69)	**<0.0001**
MF/MF	365	50 (13.7)	270 (73.97)	39 (10.68)	6 (1.64)	**<0.0001**
MF/G	33	4 (12.12)	20 (60.61)	8 (24.24)	1 (3.03)	0.51
MF/RF	221	8 (3.62)	146 (66.06)	52 (23.53)	15 (6.79)	***0.001***

Abbreviations: ppFEV_1_, percent predicted forced expiratory volume in the 1st second; HS, hypertonic saline; *Pa*, *Pseudomonas aeruginosa*, PERT, pancreatic enzyme replacement therapy; F, *F508del*; G, gating; RF, residual function, MF minimal function. Bold for p is to highlight the statistical significance.

**Table 2:** Characteristics of weight categories in paediatric patients (2–17.9 years).

Parameter	Overall n = 2015	Underweight (n = 168) (% = 8.34) n (%)	Target Weight (n = 1544) (% = 76.63) n (%)	Overweight (n = 193) (% = 9.58) n (%)	Obese (n = 110) (% = 5.46) n (%)	*p*
**Sex**						**0.03**
Male	1000	99 (9.90)	745 (74.50)	94 (9.40)	62 (6.20)	
Female	1015	69 (6.80)	799 (78.72)	99 (9.75)	48 (4.73)	
**ppFEV_1_**						**<0.0001**
<40	14	7 (50.00)	7 (50.00)	0 (0.00)	0 (0.00)	
40 to 70	104	25 (24.04)	73 (70.19)	4 (3.85)	2 (1.92)	
70 to 90	335	28 (8.36)	277 (82.69)	22 (6.57)	8 (2.39)	
90+	1082	53 (4.9)	842 (77.82)	123 (11.37)	64 (5.91)	
**Inhaled HS**						0.32
No	1002	81 (8.08)	757 (75.55)	108 (10.78)	56 (5.59)	
Yes	1013	87 (8.59)	787 (77.69)	85 (8.39)	54 (5.33)	
**Inhaled antibiotic**						0.11
No	1521	122 (8.02)	1154 (75.87)	157 (10.32)	88 (5.79)	
Yes	494	46 (9.31)	390 (78.95)	36 (7.29)	22 (4.45)	
**Diabetes**						**0.01**
No	1758	141 (8.02)	1334 (75.88)	177 (10.07)	106 (6.03)	
Yes	86	12 (13.95)	70 (81.39)	4 (4.65)	0 (0.00)	
**Pa colonization**						0.06
No	1473	112 (7.60)	1122 (76.17)	146 (9.91)	93 (6.31)	
Yes	159	15 (9.43)	128 (80.50)	14 (8.80)	2 (1.26)	
**PERT**						**<0.0001**
No	690	40 (5.80)	474 (68.70)	102 (14.78)	74 (10.72)	
Yes	1325	128 (9.66)	1070 (80.75)	91 (6.87)	36 (2.72)	
**CFTR Modulator**						**0.01**
Treated	459	37 (8.06)	370 (80.61)	40 (8.71)	12 (2.61)	
Not treated	1556	131 (8.42)	1174 (75.45)	153 (9.83)	98 (6.30)	
**Genotype**						
*F/F*	432	49 (11.34)	342 (79.17)	32 (7.41)	9 (2.08)	**0.0001**
*F/G*	40	3 (7.50)	29 (72.50)	5 (12.50)	3 (7.50)	0.85
*F/RF*	134	12 (8.95)	88 (65.67)	17 (12.69)	17 (12.69)	**0.0006**
*F/MF*	517	42 (8.12)	421 (81.43)	36 (6.96)	18 (3.48)	**0.007**
*MF/MF*	228	23 (10.09)	180 (78.95)	16 (7.02)	9 (3.95)	0.27
*MF/G*	19	1 (5.26)	15 (78.95)	2 (10.53)	1 (5.26)	0.97
*MF/RF*	130	8 (6.15)	98 (75.38)	15 (11.54)	9 (6.92)	0.59

Abbreviations: ppFEV1, percent predicted forced expiratory volume in the 1st second; HS, hypertonic saline; *Pa*, *Pseudomonas aeruginosa*, PERT, pancreatic enzyme replacement therapy; F, *F508del*; G, gating; RF, residual function, MF minimal function.

## Data Availability

The data relating to this research are available from the Italian Cystic Fibrosis Registry, and can be accessed after a reasoned request.
